# Diagnosis of Splenic Lymphoma by Endoscopic Ultrasound Guided Fine Needle Aspiration: A Case Report and Review of the Literature

**DOI:** 10.1155/2017/3602910

**Published:** 2017-04-30

**Authors:** Umar Darr, Zubair Khan, Muhammad Ali Khan, Anas Renno, Turki Alkully, Sehrish Kamal, Tariq Hammad, Yaseen Alastal, Muhammad Imran Khan, Ali Nawras

**Affiliations:** University of Toledo Medical Center, Toledo, OH, USA

## Abstract

*Introduction*. Splenic tumor is usually found as an incidental finding on CT of abdomen. Traditionally, ultrasound (US) or computed tomography (CT) guided biopsies were employed for the purpose of sampling; however they have been reported to have a complication rate of 5.3%. Endoscopic ultrasound-fine needle aspiration (EUS-FNA) has been recently utilized for the purpose of sampling splenic tumors. In literature there are 7 reported instances where splenic lymphoma was diagnosed using EUS-FNA. We present a case of follicular B cell lymphoma of the spleen diagnosed using EUS-FNA.* Case Report*. 58-year-old female presented to her primary care physician for left upper quadrant abdominal pain for one week. Physical exam was significant for left upper quadrant tenderness. Her laboratory tests were within normal limits. She underwent CT scan of abdomen which revealed approximately 5 cm × 5 cm mass in spleen. EUS-FNA of the spleen revealed a large hypoechoic, heterogeneous, well-demarcated mass measuring 54.7 mm × 43.0 mm. Fine needle aspiration was performed, and the sample was submitted for cytology and flow cytometry. Flow cytometry revealed a lambda monotypic population of B cells displaying dim CD19 and CD10. Diagnosis of B cell non-Hodgkin low grade follicular lymphoma was made.* Conclusion*. Endoscopic ultrasound with fine needle aspiration is a very rare but safe, reliable method of diagnosis of splenic lymphomas.

## 1. Introduction

Splenic tumor is seldom encountered in clinical practice and usually it is found as an incidental finding on CT scan of abdomen. Lymphoma has been reported as the most common tumor involving the spleen [1]. Traditionally, ultrasound (US) or computed tomography (CT) guided biopsies were employed for the purpose of sampling. However, they have been reported to have a complication rate of 5.3% [1]. Endoscopic ultrasound-fine needle aspiration (EUS-FNA) has been recently utilized for the purpose of sampling splenic tumors. In English literature there are only 8 reported instances where primary splenic lymphoma was diagnosed using EUS-FNA (Table 1). We, hereby, present a case of follicular B cell lymphoma of the spleen diagnosed using EUS-FNA and supported by Rapid On-Site Evaluation (ROSE), cytology, and flow cytometry.

## 2. Case Presentation

A 58-year-old female presented to her primary care physician for left upper quadrant abdominal pain of one week's duration. Pain was not associated with nausea, vomiting, hematemesis, hematochezia, or any changes in bowel movements. She had a past surgical history of cholecystectomy and hysterectomy. Physical exam was only significant for mild left upper quadrant tenderness. Her liver function tests, amylase, and lipase were all within normal limits. She underwent CT scan of abdomen, which revealed approximately 50 mm × 50 mm mass in spleen. She underwent EUS-FNA at our facility to sample the splenic mass.

**Figure 1:**
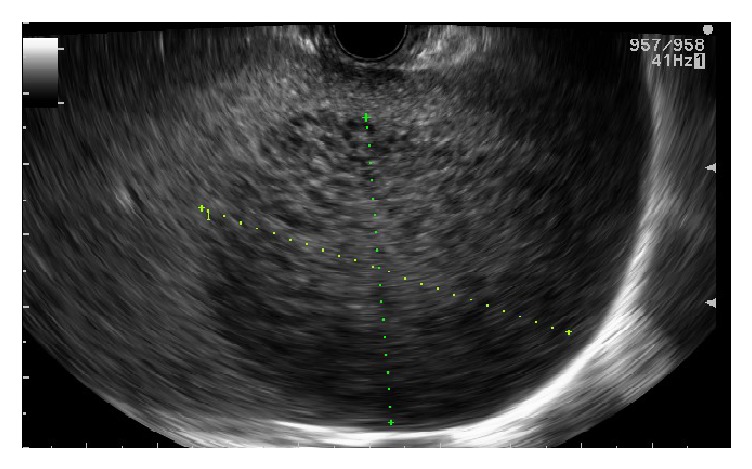
EUS revealing large hypoechoic, heterogeneous, well-demarcated mass measuring 54.7 mm × 43.0 mm.

The endosonographic examination of abdominal aorta, celiac axis, and pancreas (head, body, and tail) was unremarkable. The examination of the spleen revealed a large hypoechoic, heterogeneous, well-demarcated mass measuring 54.7 mm × 43.0 mm (Figure 1). The mass had a distinct border in contrast to the surrounding splenic tissue. The rest of the examination of the perigastric area was unremarkable. The splenic mass underwent fine needle aspiration 4 times using a 25-gauge needle. Rapid On-Site Evaluation (ROSE) was performed and the sample was submitted for cytology and flow cytometry. No procedure related complications were noted

Cytology revealed a monomorphic population of lymphoid cells of small size, admixed with a minor proportion of medium sized lymphocytes. Flow cytometry revealed a lambda monotypic population of B cells displaying dim CD19 and CD10. The neoplastic lymphoid cells were negative for CD5, CD11c, and CD103. Hence the diagnosis of B cell non-Hodgkin lymphoma (low grade follicular lymphoma) was made. The patient was subsequently referred to Oncology for management of disease.

## 3. Discussion

Primary splenic lymphoma is considered an exceptionally rare cause of splenomegaly and its reported incidence is less than 1% [2]. Despite being rare, it is imperative not only to differentiate lymphoma from benign splenic lesions but also to classify the type of lymphoma so as to initiate appropriate treatment. Percutaneous ultrasound and CT guided biopsies have been conventionally done for such lesions. Barone et al. [1] reported that such procedures are associated with risks of hemoperitoneum, pneumothorax, subacute bleeding, and subcapsular hematomas. One more drawback of percutaneous ultrasound is that visualization of spleen may be affected by surrounding structures. Also, false positive rates were higher in lymphoma patients and were not affected by sampling techniques.

On the contrary, spleen can be visualized in great detail through the gastric wall using EUS. Fritscher-Ravens et al. [3] first reported the use of EUS-FNA for diagnosing splenic lesions. They used EUS-FNA in 12 patients when US or CT guided biopsies were inconclusive, were dangerous to perform, or could not be performed due to size and location of lesion. 10 out of 12 patients were correctly diagnosed and of them two were found to have Hodgkin lymphoma of the spleen.

Another study combined EUS-FNA with flow cytometry to diagnose splenic lesions and correctly identified two patients with splenic lymphomas. However, in this study there was one false negative result, which was later on confirmed to be non-Hodgkin lymphoma after surgery [4]. Iwashita et al. [5] reported the effective usage of EUS-FNA with a 19-gauge needle in diagnosing splenic lymphomas. This series is comprised of five patients with splenic lesions of which two were diagnosed to have lymphomas, 2 have sarcoidosis, and 1 has pseudotumor of spleen.

Although there are cases that support splenic EUS guided biopsy as a safe modality, other studies have supported alternative means. In one multicenter study of image guided biopsy the complication rate was 5.2%, but the rate of major adverse events was still only 0.75% [6]. A meta-analysis of similar modality involving 9 studies found a major complication rate of 2.2% and if an 18-gauge needle or smaller was used, major complication rate was 1.3% [7]. Other recent studies of percutaneous image guided biopsy of focal splenic lesions were approximately 1% or 0% [8, 9], but these were with needles with a diameter of 18 gauges or less. Up until now, there is no study that directly compares efficacy, accuracy, and safety of image guided percutaneous biopsy versus EUS guided biopsy. There is also some question regarding the diagnostic accuracy of EUS-FNA in comparison of malignant lymphoma versus lymphoma recurrence, which is lower [10, 11]. In a study by Pugh et al., 14 cases of deep-seated lymphoma (2 involving the spleen) EUS-FNA missed the diagnosis of a high-grade large B cell lymphoma of the spleen [12]. Therefore EUS-FNA is one appropriate efficient and safe tool for sampling of solid splenic lesions; however other image guided techniques and access routes should be chosen individually taking into account patient characteristics, risk assessment, suspected diagnosis, and experience of the endoscopist.

In this review we have compiled all the reported cases of primary splenic lymphomas diagnosed using EUS-FNA (Table 1). Gender distribution was equal and mean age ± SD was 62 ± 12 years. In all cases lymphomas of spleen appear as hypoechoic lesions. No major procedure related complications were reported in any of the cases. We used the 25-gauge needle in our case and were able to diagnose low grade follicular lymphoma on EUS-FNA supported by Rapid On-Site Evaluation, cytology, and flow cytometry.

## 4. Conclusion

EUS is a safe and effective modality for diagnosing primary splenic lymphomas.

## Figures and Tables

**Table 1:** Splenic lymphoma cases and associated findings via EUS-FNA.

Ref.	Age/gender	Size	EUS features	FNA cytology/histology	FNA cytometry	Final diagnosis
[3]	41/male	28 mm	Hypoechoic	Hodgkin's lymphoma	Not done	Hodgkin's lymphoma
[3]	60/female	16 mm	Hypoechoic	Hodgkin's lymphoma	Not done	Hodgkin's lymphoma
[5]	69/female	66 × 46 mm	Hypoechoic, heterogeneous, sharp borders	Diffuse large B cell lymphoma	Monoclonal B cell	Diffuse large B cell lymphoma
[5]	67/male	53 × 45 mm	Hypoechoic, heterogeneous, unclear border	Follicular B cell lymphoma	Monoclonal B cell	Follicular B cell lymphoma
[4]	54/female	25 × 26	Hypoechoic, sharp border	Non-Hodgkin's lymphoma	Supported diagnosis	Non-Hodgkin's lymphoma
[4]	82/male	Not mentioned	Hypoechoic, sharp border	Large B cell lymphoma	Supported diagnosis	Large B cell lymphoma
[13]	66/male	120 × 110 mm	Hypoechoic	Diffuse large B cell lymphoma	Supported diagnosis	Diffuse large B cell lymphoma
[14]	51/male	50 × 39 mm	Hypoechoic	Diffuse large B cell lymphoma	Supported diagnosis	Diffuse large B cell lymphoma
Our case	58/female	54 × 43 mm	Hypoechoic, heterogeneous, sharp border	Follicular B cell lymphoma	Supported diagnosis	Follicular B cell lymphoma
